# Distinct Methylphenidate-Evoked Response Measured Using Functional Near-Infrared Spectroscopy During Go/No-Go Task as a Supporting Differential Diagnostic Tool Between Attention-Deficit/Hyperactivity Disorder and Autism Spectrum Disorder Comorbid Children

**DOI:** 10.3389/fnhum.2019.00007

**Published:** 2019-02-08

**Authors:** Stephanie Sutoko, Yukifumi Monden, Tatsuya Tokuda, Takahiro Ikeda, Masako Nagashima, Masashi Kiguchi, Atsushi Maki, Takanori Yamagata, Ippeita Dan

**Affiliations:** ^1^Center for Exploratory Research, Research & Development Group, Hitachi, Ltd., Saitama, Japan; ^2^Department of Pediatrics, Jichi Medical University, Shimotsuke, Japan; ^3^Department of Pediatrics, International University of Health and Welfare Hospital, Nasushiobara, Japan; ^4^Research and Development Initiatives, Applied Cognitive Neuroscience Laboratory, Chuo University, Tokyo, Japan; ^5^Center for Development of Advanced Medical Technology, Jichi Medical University, Shimotsuke, Japan

**Keywords:** comorbid spectrum, attention deficit/hyperactivity disorder, autism spectrum disorder, near infra-red spectroscopy, differential diagnostic tool, inhibitory task-evoked activation, methylphenidate

## Abstract

Attention deficit/hyperactivity disorder (ADHD) has been frequently reported as co-occurring with autism spectrum disorder (ASD). However, ASD-comorbid ADHD is difficult to diagnose since clinically significant symptoms are similar in both disorders. Therefore, we propose a classification method of differentially recognizing the ASD-comorbid condition in ADHD children. The classification method was investigated based on functional brain imaging measured by near-infrared spectroscopy (NIRS) during a go/no-go task. Optimization and cross-validation of the classification method was carried out in medicated-naïve and methylphenidate (MPH) administered ADHD and ASD-comorbid ADHD children (randomized, double-blind, placebo-controlled, and crossover design) to select robust parameters and cut-off thresholds. The parameters could be defined as either single or averaged multi-channel task-evoked activations under an administration condition (i.e., pre-medication, post-MPH, and post-placebo). The ADHD children were distinguished by significantly high MPH-evoked activation in the right hemisphere near the midline vertex. The ASD-comorbid ADHD children tended to have low activation responses in all regions. High specificity (86 ± 4.1%; mean ± SD), sensitivity (93 ± 7.3%), and accuracy (82 ± 1.6%) were obtained using the activation of oxygenated-hemoglobin concentration change in right middle frontal, angular, and precentral gyri under MPH medication. Therefore, the significantly differing MPH-evoked responses are potentially effective features and as supporting differential diagnostic tools.

## Introduction

Neurodevelopmental disorders are characterized as neurological conditions with impaired development in behavior, language, learning, motor, and social functions with an early onset of symptoms in infancy, childhood, or adolescence (Harris, 2014). For nearly two decades, the prevalence of neurodevelopmental disorders has increasingly reached over 13% (Merikangas et al., 2010; Boyle et al., 2011; Lawrence et al., 2015). Attention deficit/hyperactivity disorder (ADHD) and autism spectrum disorder (ASD) are among the most prevalent neurodevelopmental disorders. The prevalence rate of ADHD has steadily increased from 6.9 to 10.2% (1999–2013) (Akinbami et al., 2011; Hill et al., 2015) while that of ASD has more than doubled from 0.67 to 1.46% within 12 years (2000–2012) (Christensen et al., 2016). ADHD is characterized by age-inappropriate behaviors of inattention and hyperactivity/impulsivity. ADHD symptoms reportedly persist into adulthood (i.e., older than 18 years old) by more than 50% of cases (Kessler et al., 2006; Bruxel et al., 2014; Visser et al., 2014), causing adverse impairments of academic and work performance, social functioning, and overall quality of life (QoL) (Matza et al., 2005; Asherson et al., 2012; Brod et al., 2012). ASD symptoms are categorized by social and communication impairments and by restricted interest and repetitive behaviors. According to the Diagnostic and Statistical Manual of Mental Disorders 4th Edition (DSM-IV), ADHD and ASD are defined as completely different disorders without any comorbid interpretation. However, it has been consistently reported that both ADHD and ASD symptoms co-occur (Reiersen and Todd, 2008). Thirty-to-fifty percent of ASD-diagnosed individuals exhibit elevated ADHD symptoms (Goldstein and Schwebach, 2004; Gadow et al., 2006; Lee and Ousley, 2006), while two-thirds of ADHD-diagnosed individuals express autism symptoms (Mulligan et al., 2009).

The recently published DSM-5 started to adopt comorbidity between ASD and ADHD (American Psychiatric Association, 2013). Not only allowing the comorbidity, the DSM-5 also redefined the diagnostic guidelines for both ADHD and ASD. The major change of ADHD diagnosis is the age-of-onset symptom from 7–12 years old. Meanwhile, Asperger’s disorder and pervasive developmental disorder not otherwise specified (PDD-NOS) previously labeled and diagnosed as two distinctive disorders are specified under the ASD term according to the DSM-5. The relationship between ADHD and ASD has been extensively studied, resulting in the proposal of three comorbidity scenarios: (1) impulsivity leading to difficulties in understanding social information, (2) hyperactivity connected to stereotypic and repetitive behavior, and (3) a pairwise pathway between inattention, difficulties in understanding social information, and verbal IQ (Sokolova et al., 2017). DSM-5 significantly increased the prevalence rate from 7.38% (DSM-IV) to 10.84% due to the extension of the age-of-onset criterion particularly in inattentive symptoms (Vande Voort et al., 2014). This suggests that there is an increasing interest and awareness regarding understanding pathophysiological mechanisms, enabling treatment for not only ADHD or ASD but also comorbid ADHD-ASD.

Prior to DSM-5, translating the symptomatic features to ADHD/ASD diagnosis was not easy. It has been reported that there are five times the number of discrepancies in the evaluation of ADHD prevalence between DSM-IV and the International Statistical Classification of Diseases and Related Health Problems 10th Revision (ICD-10) (Döpfner et al., 2008; Adornetto et al., 2012). The diagnosis requires longitudinal examination that involves subjective monitoring and evaluation from multiple respondents (e.g., parents and teachers) (Soma et al., 2009). Similar issues also occur in assessing and predicting the therapeutic response. To manage pathophysiological heterogeneity, behavioral treatment and drug administration have traditionally been carried out on a trial-and-error basis, which is rather inefficient. Hence, recent studies have attempted to define the objective and measurable biological markers (biomarkers) for diagnosis, pathogenic progress, and pharmacological impact, preferably at subclass levels.

Past 20 years have witnessed significant advancements in neuroimaging technology (Bandettini, 2012; Boas et al., 2014). Accordingly, the possibility of neuroimaging for psychosis biomarkers including ADHD and ASD has being studied extensively (Hager and Keshavan, 2015). Among several neuroimaging techniques, functional near-infrared spectroscopy (fNIRS) makes evaluation of infant and children feasible (Peña et al., 2003; Sugiura et al., 2011; Monden et al., 2012a; Aslin et al., 2015; Soltanlou et al., 2018) because it is non-invasive, has high tolerability of body motion and little confinement, and is quiet (Hoshi and Michael, 2005; Cui et al., 2011; Koike et al., 2013). fNIRS measures the change in cerebral hemodynamics, which is closely related to brain metabolic activity (Maki et al., 1995). Functional magnetic resonance imaging (fMRI) also adopts the concept of brain hemodynamic response, making it advantageous regarding spatial resolution over fNIRS. However, the successful fMRI measurement rate (50–70%) is behind that of fNIRS, especially for young disordered children (i.e., >6 years old) due to motion artifacts and lack of compliance (Durston et al., 2003; Yerys et al., 2009; Monden et al., 2015). Therefore, fNIRS measurement is apparently more practical than fMRI for clinical applications.

The differences between typically developing (TD) control and ADHD patients (e.g., children, adolescents, adults) were observed using fNIRS measurement of task-related performances such as inhibition control (Monden et al., 2012a; Ishii-Takahashi et al., 2014), attention response (Nagashima et al., 2014c), verbal fluency (Matsuo et al., 2014), and facial recognition (Ichikawa et al., 2014). ADHD children significantly present lower oxygenated hemoglobin level at regions of interest (ROIs) than TD children, which may correspond to brain inactivation. ROIs depend on the association between the task and functional brain regions, for example, the right inferior frontal gyrus/middle frontal gyrus (IFG/MFG) in inhibition control tasks and both right IFG/MFG and parietal in attention response tasks (Monden et al., 2012b; Nagashima et al., 2014a,b). By using a single biomarker of oxygenated hemoglobin level (i.e., brain activation index), diagnostic analyses were developed, resulting in 78.8–85% accuracy (Ishii-Takahashi et al., 2014; Monden et al., 2015).

Functional near-infrared spectroscopy has also been used to investigate three domains in ASD, i.e., (1) non-social deficiencies, (2) atypical connectivity, and (3) social deficiencies (Liu et al., 2017). Various paradigms related to cognitive and social skills have been performed simultaneously. The brain activation in TD and ASD children depended on tasks; either ASD children have less brain activation [e.g., inhibition go/no-go (Xiao et al., 2012; Ikeda et al., 2018b), own-face recognition (Kita et al., 2011), gaze recognition (Ikeda et al., 2018a), expression of person’s mental state (Iwanaga et al., 2013)] than TD children or similar brain activation [e.g., Stroop (Xiao et al., 2012; Yasumura et al., 2014) and expression of an object’s characteristics (Iwanaga et al., 2013)] to TD children in mostly the (right) prefrontal cortex (PFC). Li and Yu (2016) conducted a classification analysis (*k*-means clustering; 83.3% accuracy) between young ASD and TD children. ASD children presented a weak efficient network between the right PFC and other regions (left PFC and bilateral temporal cortex) compared to TD children while watching a cartoon. In resting state, ASD children presented weaker bilateral functional connectivity and stronger fluctuation magnitude (oxygenated and deoxygenated hemoglobin) than TD children (Li et al., 2016). ASD-TD classification using support vector machines (SVMs) results in high sensitivity (81.6%) and specificity (94.6%).

In our recent study, we reported the significant difference in neurofunctional pathology for inhibition control in ADHD and ASD-comorbid ADHD children evaluated using fNIRS (Tokuda et al., 2018). The ADHD and ASD-comorbid ADHD children distinctly responded to methylphenidate (MPH) medication in terms of brain-activation patterns instead of showing a significantly improved ADHD symptomatic scale. Despite the favorable outcomes, the diagnostic interpretation of ASD-comorbid ADHD has not been addressed. Therefore, for the current study, we investigated suitable biomarkers for features to differentiate between individual ADHD and ASD-comorbid ADHD children. We hypothesized that different MPH-elicited responses are the main characteristics of biomarkers contributing to high specificity, sensitivity, and accuracy. We believe that an effective biomarker plays a significant role as a supporting tool for more accurate and efficient differential diagnosis between ADHD and ASD-comorbid ADHD children.

## Materials and Methods

### Subjects and Experimental Design

The dataset used in this study was obtained from the experimental data previously reported by Tokuda et al. (2018). Thirty-two medication-naïve and right-handed children diagnosed with ADHD based on the DSM-5 participated in this study. The handedness was evaluated based on the Edinburgh Handedness Questionnaire (Oldfield, 1971). Parents were asked to complete the questionnaire for their children. A questionnaire item (i.e., striking a match) was excluded due to less applicability for children activity (Hill and Khanem, 2009). Twenty-one children (7.8 ± 1.7 years old) presented only ADHD symptoms while 11 children (8.2 ± 2.1 years old) also presented ASD symptoms based on what the DSM-5 refers to as ASD-comorbid ADHD children. Both groups, i.e., ADHD and ASD-comorbid ADHD, were age- and gender-matching. However, the full scale intelligence quotient (FS-IQ) of the groups did not match. The FS-IQ scores of the ASD-comorbid ADHD group (103.2 ± 14.5) were significantly higher [*t*(30) = 2.08, *p* < 0.05, Cohen’s *d* = 0.77] than those of the ADHD group (92.8 ± 12.9). The FS-IQ of all subjects were assessed using the Wechsler Intelligence Scale of Children Third (WISC-III) or Forth (WISC-IV). All subjects provided oral consent, and written consent was obtained from the parents of all subjects according to the latest version of the Declaration of Helsinki. The study was approved by the Ethics Committees of Jichi Medical University Hospital and the International University of Health and Welfare. The collaboration among Jichi Medical University Hospital, the International University of Health and Welfare, and Hitachi, Ltd. was reviewed by an internal board at Central Research Laboratory, Hitachi, Ltd. Technical problem (e.g., data saving) unexpectedly occurred and affected data (i.e., two behavioral performance data and an fNIRS measurement data) were excluded in the further analysis.

**Figure 1:**
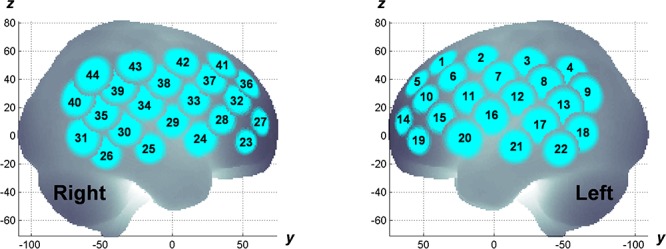
Spatial registration of fNIRS channels on bilateral hemispheric cortices.

The experiment was designed in a randomized, double-blind, placebo-controlled, and crossover study. MPH (18 mg) and placebo were administered in a pseudo-randomized order across subjects on different measurement days (at least 4 days apart). fNIRS measurements were conducted twice; before and after MPH or placebo administrations on a measurement day. fNIRS measurement involved using a multichannel system (ETG-4000, Hitachi Corporation, Tokyo, Japan) with dual wavelengths (695 and 830 nm) and a 10-Hz sampling rate. A 3×5 probe plane incorporated eight sources and seven detectors resulting in 22 channels. Two probe planes were placed on the head following the positioning manner as described elsewhere (Monden et al., 2012a). The probe locations were measured using a 3D digitizer and the channel locations were spatially registered to the Montreal Neurological Institute (MNI) standard brain spaces following the probabilistic registration method (Tsuzuki et al., 2007, 2012; Tsuzuki and Dan, 2014), as shown in Figure 1. The estimated MNI spaces were then labeled as LBPA40 (Shattuck et al., 2008) and Brodmann’s atlas (Rorden and Brett, 2000), as listed in Table 1. According to the channel registration, two probe planes covered bi-hemispheric lateral prefrontal and inferior parietal cortices (44 channels in total). During fNIRS measurements, subjects were asked to perform an inhibition control task called go/no-go (GNG) that follows the block-design paradigm involving six time trials for about 5 min in total. The measurement details, task design, and experimental protocol are described elsewhere (Monden et al., 2012a,b; Nagashima et al., 2014b).

**Table 1:** Spatial registration on fNIRS channels.

Channel	*x* (mm)	*y* (mm)	*z* (mm)	*SD* (σ; mm)	Macroanatomy	Probability
1	-27	36	51	14	L middle frontal gyrus	0.52
					L superior frontal gyrus	0.48
2	-47	9	55	14	L middle frontal gyrus	0.68
					L precentral gyrus	0.32
3	-59	-25	52	14	L supramarginal gyrus	0.55
					L postcentral gyrus	0.46
4	-58	-55	46	14	L angular gyrus	0.86
					L supramarginal gyrus	0.14
5	-24	55	38	13	L middle frontal gyrus	0.85
					L superior frontal gyrus	0.15
6	-46	30	42	13	L middle frontal gyrus	1.00
7	-60	-4	41	14	L precentral gyrus	0.52
					L postcentral gyrus	0.46
					L supramarginal gyrus	0.02
8	-67	-36	38	14	L supramarginal gyrus	0.96
					L angular gyrus	0.04
9	-59	-65	30	15	L angular gyrus	0.78
					L middle occipital gyrus	0.20
					L supramarginal gyrus	0.02
					L middle temporal gyrus	0.01
					L superior temporal gyrus	0.00
10	-42	48	26	12	L middle frontal gyrus	0.99
					L inferior frontal gyrus	0.01
11	-58	17	27	14	L inferior frontal gyrus	0.42
					L precentral gyrus	0.33
					L middle frontal gyrus	0.25
12	-68	-17	27	14	L postcentral gyrus	0.49
					L supramarginal gyrus	0.40
					L superior temporal gyrus	0.11
13	-67	-50	21	15	L superior temporal gyrus	0.30
					L supramarginal gyrus	0.29
					L angular gyrus	0.25
					L middle temporal gyrus	0.16
14	-34	64	11	12	L middle frontal gyrus	0.95
					L inferior frontal gyrus	0.05
15	-54	37	13	13	L inferior frontal gyrus	0.85
					L middle frontal gyrus	0.15
16	-65	2	13	15	L precentral gyrus	0.51
					L postcentral gyrus	0.29
					L superior temporal gyrus	0.14
					L inferior frontal gyrus	0.06
17	-70	-32	8	15	L superior temporal gyrus	0.63
					L middle temporal gyrus	0.38
18	-64	-62	2	15	L middle temporal gyrus	0.72
					L inferior temporal gyrus	0.16
					L middle occipital gyrus	0.09
					L angular gyrus	0.03
					L inferior occipital gyrus	0.00
19	-46	54	-3	11	L inferior frontal gyrus	0.82
					L lateral orbitofrontal gyrus	0.18
					L middle frontal gyrus	0.01
20	-56	21	-2	16	L inferior frontal gyrus	0.56
					L superior temporal gyrus	0.31
					L lateral orbitofrontal gyrus	0.08
					L precentral gyrus	0.05
21	-70	-15	-7	13	L middle temporal gyrus	0.77
					L superior temporal gyrus	0.23
22	-68	-47	-10	14	L middle temporal gyrus	0.60
					L inferior temporal gyrus	0.40
23	50	52	-4	10	R inferior frontal gyrus	0.82
					R lateral orbitofrontal gyrus	0.17
					R middle frontal gyrus	0.01
24	58	19	-2	15	R inferior frontal gyrus	0.45
					R superior temporal gyrus	0.34
					R precentral gyrus	0.15
					R lateral orbitofrontal gyrus	0.03
					R middle temporal gyrus	0.02
25	72	-16	-9	12	R middle temporal gyrus	0.79
					R superior temporal gyrus	0.21
26	69	-48	-12	14	R middle temporal gyrus	0.54
					R inferior temporal gyrus	0.46
27	40	63	10	11	R middle frontal gyrus	0.76
					R inferior frontal gyrus	0.24
28	58	35	12	12	R inferior frontal gyrus	1.00
29	67	0	11	14	R postcentral gyrus	0.39
					R superior temporal gyrus	0.35
					R precentral gyrus	0.26
					R inferior frontal gyrus	0.01
30	73	-33	5	14	R middle temporal gyrus	0.53
					R superior temporal gyrus	0.47
31	63	-63	0	16	R middle temporal gyrus	0.39
					R middle occipital gyrus	0.31
					R inferior temporal gyrus	0.30
					R angular gyrus	0.00
32	48	46	26	13	R middle frontal gyrus	0.63
					R inferior frontal gyrus	0.38
33	62	15	26	14	R precentral gyrus	0.74
					R inferior frontal gyrus	0.24
					R middle frontal gyrus	0.02
					R postcentral gyrus	0.01
34	70	-19	24	15	R supramarginal gyrus	0.41
					R postcentral gyrus	0.31
					R superior temporal gyrus	0.27
					R angular gyrus	0.02
35	68	-50	18	15	R middle temporal gyrus	0.39
					R angular gyrus	0.31
					R superior temporal gyrus	0.24
					R supramarginal gyrus	0.06
					R middle occipital gyrus	0.01
					R inferior temporal gyrus	0.00
36	31	53	37	14	R middle frontal gyrus	0.97
					R superior frontal gyrus	0.03
37	51	27	40	14	R middle frontal gyrus	0.84
					R inferior frontal gyrus	0.11
					R precentral gyrus	0.05
38	65	-6	39	14	R postcentral gyrus	0.49
					R supramarginal gyrus	0.27
					R precentral gyrus	0.24
39	68	-38	36	15	R supramarginal gyrus	0.65
					R angular gyrus	0.29
					R superior temporal gyrus	0.06
40	58	-68	27	16	R angular gyrus	0.62
					R middle occipital gyrus	0.37
					R middle temporal gyrus	0.01
41	34	34	50	14	R middle frontal gyrus	0.88
					R superior frontal gyrus	0.12
42	52	6	52	15	R precentral gyrus	0.55
					R middle frontal gyrus	0.36
					R postcentral gyrus	0.09
43	64	-26	51	15	R supramarginal gyrus	0.98
					R postcentral gyrus	0.02
44	60	-56	44	16	R angular gyrus	0.95
					R supramarginal gyrus	0.05

*x is defined as 0 at the midline vertex (nasion-to-inion). y is defined as 0 at the lateral vertex of left to right preauricular points. z is defined as 0 at the head circumference plane*.

Parents evaluated their children on a Japanese version of the ADHD Rating Scale-IV (ADHD-RS-IV) (Yamazaki, 2003) before and after 1 month of MPH administration. According to Tokuda et al.’s (2018) results corresponding to this dataset, MPH resulted in significant improvement as evidenced from low ratings on the ADHD-RS IV for all characteristics (i.e., inattention and hyperactivity) for both ADHD and ASD-comorbid ADHD groups. The relationship between symptomatic improvement and brain imaging would be an interesting discussion, but our study was focused on differential diagnostic biomarkers instead of investigating pharmacological effects. Furthermore, the parents qualitatively evaluated their children on the ADHD-RS-IV; thus, we should not rule out the subjectivity factor across subjects. Therefore, we put aside the qualitative symptomatic variable.

### Behavioral Performance Data

There were five features extracted from behavioral performance data – (1) accuracy of go response during the baseline period (i.e., 1 – omission error), (2) accuracy of go response during the stimulus period, (3) accuracy of no-go response during the stimulus period (i.e., 1 – commission error), (4) response time of correct go response during the baseline period, and (5) response time of correct go response during the stimulus period. The differences between the ADHD and ASD-comorbid ADHD groups were then statistically evaluated (two-sample *t*-test) in each condition (i.e., first measurement, post-MPH, and post-placebo administrations).

### Analysis of fNIRS Data

Signal preprocessing was carried out on the MATLAB-based software Platform for Optical Topography Analysis Tools (POTATo, Hitachi Ltd., Research and Development) (Sutoko et al., 2016). The optical density data were initially converted into the product of hemoglobin concentration change and optical path length (Maki et al., 1995; Koizumi et al., 2003; Katagiri et al., 2010) defined as ΔC_O2Hb_, ΔC_HHb_, and ΔC_Hb-total_ (in mM⋅mm) based on the modified Beer-Lambert law (Delpy et al., 1988; Maki et al., 1995). Forty-four continuous ΔC signals were preprocessed with first-degree polynomial fitting and band-pass filtering (0.01–0.8 Hz cut-off) to remove baseline drift and cardiac pulsation. Channel-wise signals were then cut according to the task trial including 13 s of baseline, 24 s of stimulus, and 13 s of post-stimulus. Therefore, six trial-wise signals were obtained from each channel. As previously reported, trial signals affected by motion artifacts with sudden, obvious, and discontinuous noise were rejected based on visual examination by two raters. We developed an algorithm to automatically computerized noise detection and rejection (Sutoko et al., 2018). This algorithm is based on the inter-trial correlation summation. Noisy trial signals apparently had low temporal correlations with other trial signals. Therefore, the noisy inter-trial correlation summation would be low compared to noise-free ones. The trial signals having (low) outlier inter-trial summation would be rejected. Non-parametric outlier assessment (i.e., Tukey’s fences) was carried out based on the interquartile range and constant (*k*), which was determined by the optimization of the rejection accuracy between visual examination and algorithm application. The optimum *k* was found to be 3 resulting in 96.1% rejection accuracy. We eliminated channel-wise data having more than two trial signals rejected. The noise-free trial signals were then fitted by the averaged amplitude of the 10-s baseline for each trial, channel, and signal type (O_2_Hb, HHb, Hb-Total).

### fNIRS Data Characteristics

Our previous results indicated the prominent roles of the right MFG and IFG in the GNG task (Monden et al., 2012a,b; Nagashima et al., 2014b; Tokuda et al., 2018). Therefore, in the current study, we focused on the right hemisphere. The characteristics of brain activation were evaluated in both groups. Brain activation was defined as the channel-wise averaged amplitude of ΔC_O2Hb_ and ΔC_HHb_ from 4 s after onset to the end of stimulus across trials. Brain activation was initially observed from group analyses (ADHD and ASD-comorbid ADHD) at the first measurement (before any administrations), after MPH administration, and after placebo administration as the exploratory data analysis (one-sample *t*-test). The differences between the ADHD and ASD-comorbid ADHD groups were then statistically evaluated (two-sample *t*-test).

### Optimization of Individual Classification and Cross-Validation

The significant differences between the two groups would be hints of effective features. Consequently, individual classification was optimized using only the significantly differing characteristics. Extensive optimization was done for each characteristic (e.g., activation of ΔC_O2Hb_ and ΔC_HHb_ under each administration condition). Significant differences may occur in more than one channel-wise activation. Therefore, classification optimization included multiple channel selection. To avoid spurious optimization in channel selection, the significant channels were categorized on the basis of brain macroanatomy. If a channel was located between two or more regions, the spatial grouping would be determined by the higher region probability (see Table 1). A spatial-related group was represented by the average of activation channels. The use of more than one spatial group was computerized by averaging the activations across groups.

**Figure 2:**
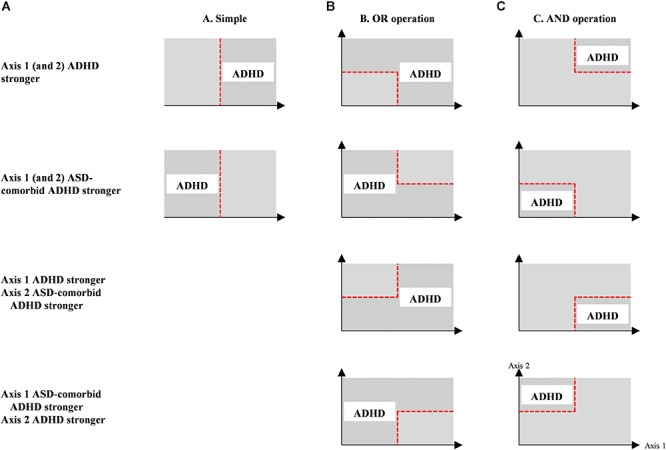
Optimization operations (i.e., simple, OR, AND operations) for individual classification between ADHD and ASD-comorbid ADHD groups using either one- (i.e., simple operation); **(A)** or two-axis (i.e., OR and AND operations; **(B,C)** feature threshold. The estimation of ADHD classification is represented by patterned areas while ASD-comorbid ADHD subjects are categorized within plain areas.

This optimization was conducted in six operations, i.e., simple, OR, AND, linear discriminant, quadratic discriminant, and SVM. The simple operation classified subjects based on the one-axis feature threshold (single or multiple channels/groups) in receiver operating characteristic (ROC) analysis. The OR and AND operations categorized subjects using the two-axis feature thresholds (single or multiple channels) with different operation between thresholds (Monden et al., 2015). Figure 2 shows the optimization operations with specific group differences (i.e., stronger activations in the ADHD group, and *vice versa*) and estimated group classification areas. Linear and quadratic discriminants and SVM operations were carried out in the two-axis feature thresholds, similar to OR and AND operations. There was no overlapping channels or groups in the two-axis feature thresholds. To confirm the robustness of feature performance, we conducted leave-one-out cross validation with 32 iterations (i.e., 31 training samples and 1 test sample). The optimum result was determined by high averaged specificity (true ADHD) – sensitivity (true ASD-comorbid ADHD) in the training data and high accuracy in the test data.

The significantly differed behavioral performance data (i.e., accuracy and response time) between ADHD and ASD-comorbid ADHD groups were also optimized for diagnostic features using similar operations. Accuracy and response time features were classified separately without any combination across features. The combination for an axis may only occur within a feature – for example, the average of go response accuracy during baseline and stimulus periods. The diagnostic performances using brain and behavioral features were compared to assess the feature efficacy.

## Results

### Behavioral Performance in Inhibition Control

**Figure 3:**
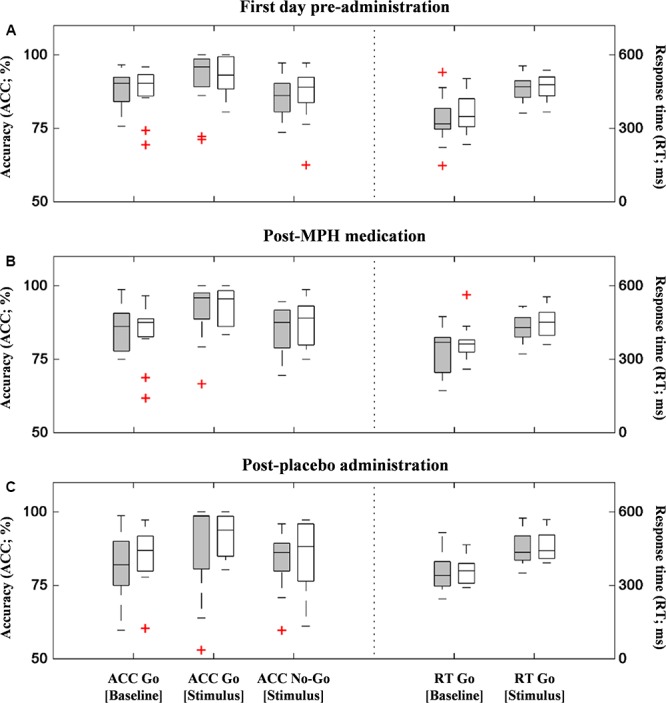
Behavioral performances (accuracy – left *y*-axis and response time – right *y*-axis) of ADHD (gray-filled boxplots) and ASD-comorbid ADHD (i.e., void-filled boxplots) groups in the first measurement **(A)**, post-MPH **(B)**, and post-placebo **(C)** administrations. There was no significant difference between two groups in any performance data and administration conditions.

Figure 3 shows the boxplots of behavioral performance data for ADHD and ASD-comorbid ADHD groups in each administration condition. There was no significant difference (i.e., two-sample *t*-test) between ADHD and ASD-comorbid ADHD groups in any behavioral performance features. Multivariate ANOVA (i.e., groups, features, administration conditions) and *post hoc* analysis were further performed showing the significances of performance features and the insignificances of group and administration conditions. The accuracy of go response was increased [*F*(2,271) = 5.67, *p* < 0.01] in the stimulus period in parallel with slower reaction time [*F*(1,186) = 87.14, *p* < 0.001] compared to the baseline period. This may suggest that the behavioral performance was likely influenced by the task paradigm *per se* rather than group or administration condition.

### Exploratory Analysis of Group Characteristics

**Figure 4:**
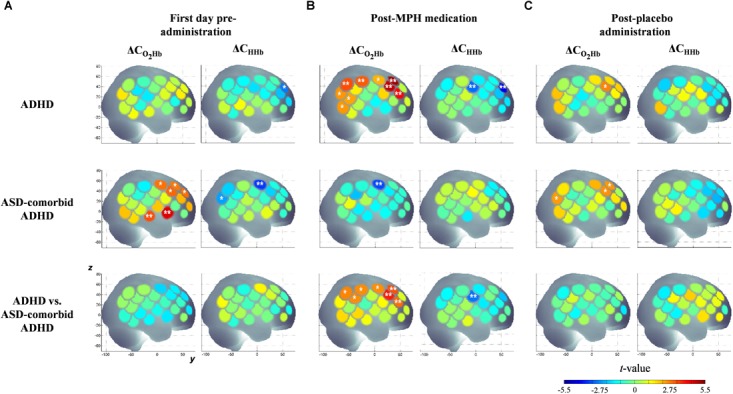
Statistical *t*-maps of ΔC_O2Hb_ and ΔC_HHb_ activation pattern in right hemisphere for both ADHD and ASD-comorbid ADHD children during GNG task before any administration **(A)**, after medication **(B)**, and placebo **(C)** administrations. The differences between ADHD and ASD-comorbid ADHD children were statistically examined (i.e., two-sample *t*-test). Two and single asterisks indicate channels with the significant activation by *p* < 0.01 and *p* < 0.05, respectively.

According to the activation patterns of ΔC_O2Hb_ and ΔC_HHb_ shown in Figure 4A, we found that the ADHD group showed significant decreases in ΔC_HHb_ [*t*(19) = -2.79, *p* < 0.05] in the right MFG but no significant ΔC_O2Hb_ changes on the first day of pre-administration. However, the ASD-comorbid ADHD group showed significant increase of ΔC_O2Hb_ [*t*(10) = 2.91–4.48, *p* < 0.05] in the right IFG, MFG, superior temporal gyrus (STG), and precentral gyrus (PrCG). The decrease of ΔC_HHb_ [*t*(10) = -2.38 – -3.55, *p* < 0.05] was observed in the right PrCG. There were no significant differences (i.e., two-sample *t*-test, DF = 29, *p* ≥ 0.05) between ADHD and ASD-comorbid ADHD groups in both ΔC_O2Hb_ and ΔC_HHb_ activations. After MPH administration (Figure 4B), the ADHD group presented significant increases in ΔC_O2Hb_ [*t*(20) = 2.19 – 5.14, *p* < 0.05] and decreases in ΔC_HHb_ [*t*(20) = -2.97 – -4.37, *p* < 0.05] in substantial areas of the right MFG/IFG and parts of the PrCG, postcentral gyrus (PoCG), supramarginal gyrus (SMG), angular gyrus (ANG), STG, and middle temporal gyrus (MTG). The ASD-comorbid ADHD group responded differently to MPH administration by presenting significant decreases in ΔC_O2Hb_ [*t*(10) = -3.35, *p* < 0.05] in the right PrCG. This group comparison suggested that MPH resulted in significant increases in ΔC_O2Hb_ [*t*(30) = 2.60–3.84, *p* < 0.05] and decreases in ΔC_HHb_ [*t*(30) = -2.86, *p* < 0.05] in the ADHD group. The ΔC_O2Hb_ apparently increased in most of the right MFG extending to the right PrCG, SMG, and ANG, while a significant decrease in ΔC_HHb_ was observed in a single channel (38) of the right PoCG. In post-placebo administration (Figure 4C), the ADHD group presented increases in ΔC_O2Hb_ [*t*(20) = 2.69, *p* < 0.05] in the right MFG. The ASD-comorbid ADHD group also presented significant increases in ΔC_O2Hb_ in the right MFG [*t*(10) = 2.49–2.57, *p* < 0.05] and ANG [*t*(10) = 2.44, *p* < 0.05] without observed ΔC_HHb_ significances. The differences of ΔC_O2Hb_ and ΔC_HHb_ activations were insignificantly found in this inter-group comparison. Table 2 details the statistical results of ΔC_O2Hb_ and ΔC_HHb_ activations for each group, administration condition, and region.

**Table 2:** Statistical results of brain activation analysis.

		First day pre-administration		Post-MPH administration		Post-placebo administration	
		ΔCo_2Hb_	ΔC_HH__b_	ΔCo_2Hb_	ΔC_HH__b_	ΔCo_2Hb_	ΔC_HH__b_
ADHD	IFG						
	STG						
	MTG			CH 31, 35 (*d* = 0.51 -0.54)			
	MFG		CH 36 (*d* = -0.62)	CH 32, 37, 41 (*d* = 0.91-1.15)	CH 36 (*d* = -0.98)	CH 37 (*d* = 0.59)	
	PrCG			CH 42 (*d* = 0.48)			
	PoCG				CH 38 (*d* = -0.65)		
	SMG			CH 43 (*d* = 0.69)			
	ANG			CH 40, 44 (*d* = 0.55-0.72)			

ASD-comorbid ADHD	IFG	CH 24 (*d* = 1.35)					
	STG	CH 25 (*d* = 1.00)					
	MTG						
	MFG	CH 36, 37, 41 (*d* = 0.85 -0.92)				CH 37, 41 *(d* = *0.*75-0.77)	
	PrCG	CH 42 (*d* = 0.88)	CH 42 (*d* = -1.07)	CH 42 (*d* = -1.01)			
	PoCG						
	SMG						
	ANG		CH 40 (*d* = -0.72)			CH 40 (*d* = 0.74)	

ADHD vs.	IFG						
ASD-comorbid ADHD	STG						
	MTG						
	MFG			CH 32, 37, 41 *(d* = 1.10-1.42)			
	PrCG			CH 42 (*d* = 0.97)			
	PoCG				CH 38 (*d* = -1.07)		
	SMG			CH 39, 43 (*d* = 0.97 –0.98)			
	ANG			CH 44 (*d* = 1.00)			

*d is Cohen’s effect size*.

### Optimization of Individual Features

Different from the conditions of pre-administration and post-placebo administration, the post-MPH condition clearly showed significant differences between the ADHD and ASD-comorbid ADHD groups. Therefore, only the significant activations of the post-MPH administration condition were optimized for features. As shown in Figure 4, ΔC_HHb_ activation presented a single significant channel. The simple operation can be solely carried out under these conditions. Meanwhile, ΔC_O2Hb_ activation significantly differed in the broad area around the midline vertex. Seven significant channels (Figure 4) were grouped into four spatial groups, i.e., right MFG (channels 32, 37, 41), right PrCG (channel 42), right SMG (channels 39, 43), and right ANG (channel 44). All optimization operations were thus applicable for ΔC_O2Hb_ activation.

Table 3 summarizes the feature performances. The ΔC_O2Hb_ activation was a better feature index in majority compared to ΔC_HHb_ activation, as shown by the higher summation of specificity and sensitivity. This may be related to the more prominent significance of channel-wise ΔC_O2Hb_ activation between the ADHD and ASD-comorbid ADHD groups. To statistically evaluate the classification performances, univariate ANOVA and *post hoc* analysis were carried out across operations and features. The SVM operation with features of right MFG-PrCG (channels 32, 37, 41, and 42; axis 1) and right SMG-ANG (channel 39, 43, and 44; axis 2) significantly presented the highest specificity (94 ± 3.4%). The highest sensitivity (100%) was offered by four combinations of feature-operation (Table 3). Furthermore, the preeminent summation of specificity and sensitivity was presented by the OR and SVM operations with the features of right MFG-ANG (channels 32, 37, 41, and 44; axis 1) and right PrCG (channel 42; axis 2). Even though the OR operation presented the maximum sensitivity and the SVM operation apparently showed the well-balance specificity- sensitivity performance, the linear discriminant operation with the same features provided the highest cross validation accuracy (84%). This should be noted that the sample number was currently limited, three-percent of accuracy difference was only caused by five and six misclassifications for the linear discriminant and OR/SVM operations, respectively. Even though it was difficult to determine the best performed operation, we could confirm that right MFG-ANG and right PrCG were optimum and relatively robust with all optimization operations compared to other spatial groups. By calculating the pooled variance among operations (OR, linear discriminant, quadratic discriminant, and SVM) in the optimum spatial groups, specificity, sensitivity, and accuracy were 86 ± 4.1%, 93 ± 7.3%, 82 ± 1.6%, respectively. Because there was no significantly observed difference of behavioral performance between ADHD and ASD-comorbid ADHD groups, performance characteristics were not optimized further.

**Table 3:** Summary of optimum features based on the characteristics of brain activation.

Characteristic	Condition	Optimization operation	Feature(s)	Specificity	Sensitivity	Accuracy
ΔC_O2Hb_ activation	Post-MPH	Simple	CH 32, 37, 41, 44	67 ± 1.9%	100%	72%
	Medication		CH 32, 37, 41, 42	76 ± 1.8%	91 ± 1.7%	75%
			CH 32, 37, 41, 42, 44	86 ± 1.4%	82 ± 2.3%	72%
		OR operation	CH 32, 37, 41, 44 (Axis 1) CH 42 (Axis 2)	81 ± 1.6%	100%	81%
			CH 32, 37, 41, 42 (Axis 1) CH 39, 43, 44 (Axis 2)	76 ± 1.8%	91 ± 1.7%	63%
		AND operation	CH 32, 37, 41, 44 (Axis 1) CH 42 (Axis 2)	67 ± 1.9%	100%	59%
			CH 32, 37, 41, 42 (Axis 1) CH 39, 43, 44 (Axis 2)	76 ± 1.8%	100%	72%
		Linear discriminant	CH 32, 37, 41, 44 (Axis 1) CH 42 (Axis 2)	85 ± 1.8%	84 ± 4.5%	84%
			CH 32, 37, 41, 42 (Axis 1) CH 39, 43, 44 (Axis 2)	76 **±** 2.4%	80 ± 4.6%	75%
		Quadratic discriminant	CH 32, 37, 41, 44 (Axis 1) CH 42 (Axis 2)	86 ± 1.7%	94 ± 7.0%	81%
			CH 32, 37, 41, 42 (Axis 1) CH 39, 43, 44 (Axis 2)	78 **±** 4.2%	85 ± 4.3%	72%
		Support vector machine	CH 32, 37, 41, 44 (Axis 1) CH 42 (Axis 2)	91 ± 2.1%	92 ± 4.5%	81%
			CH 32, 37, 41, 42 (Axis 1) CH 39, 43, 44 (Axis 2)	94 ± 3.4%	57 ± 11%	63%
ΔC_HHb_ activation	Post-MPH medication	Simple	CH38	90 ± 1.2%	64 ± 2.9%	75%

**Figure 5:**
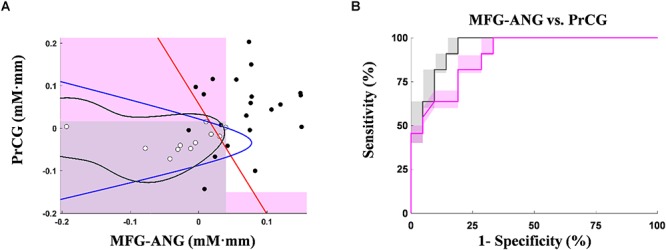
Activation coordinates for ADHD (black-dots) and ASD-comorbid ADHD (white-dots) groups using the optimum MPH-evoked response on ΔC_O2Hb_ activation in right MFG-ANG vs. right PrCG **(A)**. Shaded regions are classification areas for the ASD-comorbid ADHD group using OR (gray-patch; cut-off thresholds at 0.04 mM⋅mm and 0.016 mM⋅mm for axis 1 and 2, respectively) and AND (magenta-patch; cut-off thresholds at 0.04 mM⋅mm and –0.15 mM⋅mm for axis 1 and 2, respectively) operations. Other classification operations are presented in red-, blue-, and black-plots for linear discriminant, quadratic discriminant, and SVM, respectively. ROC graphs of leave-one-out cross-validation results with the optimum MPH-evoked response on ΔC_O2Hb_ activation in right MFG-ANG vs. right PrCG **(B)** using OR (gray-plots) and AND (magenta-plots) operations. The bold lines indicate averages of cross-validation result. The shaded regions represent the range of validation performance (minimum–to-maximum specificity and sensitivity).

**Figure 6:**
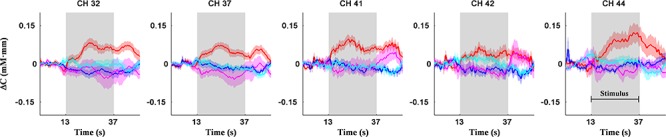
Subject-average for channel-wise ΔC_O2Hb_ (red- and magenta-plots) and ΔC_HHb_ (blue- and cyan-plots) waveforms for ADHD (*N* = 21; red- and blue-plots) and ASD-comorbid ADHD (*N* = 11; magenta- and cyan-plots) groups in right MFG (channels 32, 37, 41), right ANG (channel 44), and right PrCG (channel 42) regions. Patches around bold plots indicate standard error and gray-shaded interval is the stimulus interval of GNG task (24 s).

Figure 5 shows the activation coordinates and ROC graphs using the two-axis feature thresholds (i.e., the post-MPH ΔC_O2Hb_ activations in right MFG-ANG vs. right PrCG). By using all optimization operations, the classification spaces were also incorporated in the activation coordinate (Figure 5A). The highest specificity (91%; *N* = 21) was presented by the SVM operation (black-line) whereas the OR and AND operations (gray and magenta spaces, respectively) performed the complete classification of ASD-comorbid ADHD group (100%; *N* = 11). The activation differences were observed further in the subject-average ΔC_O2Hb_ and ΔC_HHb_ waveforms of the ADHD and ASD-comorbid ADHD groups in corresponding channels (i.e., 32, 37, 41, 42, and 44; Figure 6). The increase of MPH-evoked response on ΔC_O2Hb_ was clearly observed in the ADHD group (red-plots) compared to the ASD-comorbid ADHD group (magenta-plots). The intra-group difference on ΔC_HHb_ (blue- and cyan-plots) was insignificantly distinguished.

## Discussion

To the best of our knowledge, this is the first study showing classification based on inhibitory responses in ADHD and ASD-comorbid ADHD children since the comorbidity of ADHD and ASD has not been recognized until recently. We found distinct MPH-evoked response during the GNG task between ADHD and ASD-comorbid ADHD children. MPH medication reduced the activation in ASD-comorbid ADHD children, while ADHD children experienced positive neuromodulation after MPH administration in major areas of the right hemisphere. The applicability of MPH-evoked response as a differential group feature was optimized and cross-validated. High classification accuracy (i.e., specificity, sensitivity) in both training and test datasets suggested the advantages of our current fNIRS, inhibitory paradigm, and analysis. We believe that our findings will likely enable recognition of ASD comorbidity in ADHD children. This is one step further in the development of a clinically differential diagnostic tool that supports the standard symptom-based examination.

### Medicated-Naïve ADHD and ASD-Comorbid ADHD Group Differences in Inhibition Response

The activation in several brain regions, including the bilateral ventrolateral prefrontal cortex (VLPFC) and dorsolateral prefrontal cortex (DLPFC) (or IFG and MFG), SMA, anterior cingulate gyrus, inferior parietal and temporal lobes, caudate nucleus, and cerebellum, has been observed during performance of the GNG task (Garavan et al., 1999; Liddle et al., 2001; Menon et al., 2001; Rubia et al., 2003). The right IFG plays an particularly important role in inhibition response not only for the GNG task but also for stop-signal and other analogous tasks (Menon et al., 2001; Aron et al., 2003, 2004; Rubia et al., 2003). Inhibition response by the right IFG may specifically relate to cue recognition regardless of the involvement of inhibition output (e.g., motor response) (Hampshire et al., 2010; Aron et al., 2014). Low right IFG activation during inhibition tasks was frequently reported in medicated-naïve or medication washed-out ADHD subjects compared to TD controls (Rubia et al., 1999, 2005; Monden et al., 2012b; Nagashima et al., 2014b). We found no significant activation in the ADHD group before any administration, which is consistent with the suggested hypothesis of right IFG impairment in ADHD.

ASD patients have also been reported as having low activation compared to TD controls in the bilateral DLPFC, left VLPFC, left premotor area, left pre-supplementary motor area, and frontal pole during the inhibitory stop-signal task (Xiao et al., 2012; Ishii-Takahashi et al., 2014). However, there was a counter observation. Vara et al. (2014) evaluated the inhibitory response of ASD adolescents measured using magnetoencephalography. Compared to healthy young adults, ASD adolescents recruited large part of the frontal cortex for the inhibitory process, yet showed poor behavioral performance (Belmonte et al., 2004; Schulz et al., 2004). This suggested that the neuropathophysiology of ASD affects either low activation or impaired selective recruitment of the brain areas. Apart from ADHD children and ASD children with low activation, ASD-comorbid ADHD children showed significant right IFG/MFG ΔC_O2Hb_ activation. Chantiluke et al. (2014) hypothesized that “the comorbidity is neither an endophenocopy of the two pure disorders nor an additive pathology.”

The comparison of behavioral performance among TD controls, ADHD and ASD patients had been previously investigated. The inconsistencies were observed by either significant or null differences of inhibitory control performance. TD controls showed higher accuracy response (Monden et al., 2012b; Xiao et al., 2012; Vara et al., 2014), faster response time (Alderson et al., 2008; Xiao et al., 2012), and less variability of response time (Smith et al., 2006; Alderson et al., 2008; Hart et al., 2014; Tye et al., 2014) than ADHD/ASD children or adolescents. Meanwhile, some studies reported insignificant differences of performance parameters between TD controls and ADHD/ASD children or adolescents (Kana et al., 2007; Nagashima et al., 2014b; Ikeda et al., 2018a,b). The contrast between ADHD and ASD patients was also insignificantly distinguished (Ishii-Takahashi et al., 2014; Tye et al., 2014). Nevertheless, functional imaging results (e.g., fNIRS, fMRI, magnetoencephalography, electroencephalography) of aforementioned studies could interpret the group differences better than behavioral performances.

Sinzig et al. (2008) evaluated four groups of TD controls, ADHD, ASD, and ASD-comorbid ADHD children–adolescents in four behavioral paradigms including inhibition, sustained attention, divided attention, and alertness. The significant group effect was only shown in attentional-related paradigms; the inhibitory performances (e.g., omission and commission errors) were comparable among groups. The differences of inhibitory performance between ADHD and ASD-comorbid ADHD groups were also not pronounced in the current study; yet, the hyperactivity rating scale before MPH administration differed in both groups [*t*(30) = -2.38, *p* < 0.05]. Similar to previous results, only functional imaging results presented group-related activation patterns. This might suggest that the group differences were more feasibly observed by functional brain features than behavioral performances.

### MPH-Evoked Response in ADHD and ASD-Comorbid ADHD Groups

ASD treatment focused on co-morbidity impairment (e.g., irritability) rather than the core of ASD phenotypes (Davis and Kollins, 2012; Santosh and Singh, 2016). Due to the recent concern of co-occur ADHD-related symptoms in ASD children, ASD children are also often prescribed ADHD medication (e.g., psychostimulants and non-stimulants) (Davis and Kollins, 2012). Fifty-eight percent of ASD-comorbid ADHD children are more likely to take psychiatric medication than either ADHD- (49%) or ASD-only (34%) diagnosed children (Frazier et al., 2011). MPH has been widely used for treating ASD-comorbid ADHD children, showing symptomatic (e.g., hyperactivity and inattentive) improvement with a lower respondent rate (50%) than ADHD children (70–80%) (Greenhill et al., 1996, 2006; Frazier et al., 2011); in the current study, the MPH respondent rate was the same for both ADHD and ASD-comorbid ADHD children (81.8%; total hyperactivity – inattentive rating scale). Even though the symptomatic improvement was observed after MPH treatment, the behavioral performances (e.g., accuracy and response time) in both groups were insignificantly influenced by the administration conditions (i.e., multivariate ANOVA). Previous studies also failed to report the significant contrast between medication (e.g., MPH, atomoxetine, fluoxetine) and placebo administrations in behavioral performances (Monden et al., 2012b; Nagashima et al., 2014b; Chantiluke et al., 2015). ASD-comorbid ADHD children were reported to have low tolerability against medication dose and exhibit adverse effects (Frazier et al., 2011). These studies suggest that the effects of medication are still difficult to predict from assessing behavioral performance; therefore, neuroimaging-based monitoring is increasing in importance.

Besides relatively predictable MPH-induced activation changes in the right IFG/MFG, as described in the previous section, we should discuss activation in the parietal cortex after MPH administration. MPH has a 10 times higher affinity to dopamine than to noradrenaline (Bymaster et al., 2002), and MPH medication in non-naïve medicated ADHD children modulated the prefrontal but not parietal regions (Nagashima et al., 2014a), as the dopamine system involves the prefrontal and striatal regions (Faraone and Biederman, 1998). However, the current dataset contained data from medicated-naïve children, which might be more prone to any medication efficacy. The current findings also suggest that the (MPH) medication-related response on brain activation differs depending on the disorder. As previously described, MPH administration consistently modulated the increase in ΔC_O2Hb_ activation in ADHD children during the GNG task (Monden et al., 2012a,b). However, ASD-comorbid ADHD children presented decreases in ΔC_O2Hb_ activation after MPH administration. This could be explained by the potential difference in strategic inhibitory control; thus, the pre- and post-medicated conditions of ASD-comorbid ADHD children is in contrast with that of ADHD children. This may also be affected by multifactorial circumstances including the severity of ASD-related symptoms in ADHD children.

### MPH-Evoked Response as Differential Feature

In the neurovascular coupling theorem, the typical fNIRS activation signal is anti-correlated between ΔC_O2Hb_ and ΔC_HHb_ (Obrig et al., 2000); a significant increase in ΔC_O2Hb_ activation is simultaneous with a significant decrease in ΔC_HHb_ activation. However, we found more significant differences in ΔC_O2Hb_ activation than in ΔC_HHb_ activation, as presented in previous studies (Monden et al., 2012b; Ishii-Takahashi et al., 2014; Nagashima et al., 2014b). Insensitive and inconsistent ΔC_HHb_ regarding cerebral blood flow change had been reported (Hoshi et al., 2001; Hirasawa et al., 2014) while the ΔC_O2Hb_ response on cerebral blood flow change was more robust and profound (Hoshi et al., 2001; Strangman et al., 2002; Hoshi, 2003). Among the three administration conditions, post-MPH medication showed significant differences in terms of the size of significantly activated regions and statistical power (*p* < 0.01). Therefore, the optimum feature was obtained from the characteristics of ΔC_O2Hb_ activation after MPH intake. We conducted leave-one-out cross-validation analysis to confirm the channel selections, cut-off thresholds, and suitability of optimization operations (i.e., simple, OR, AND, linear discriminant, quadratic discriminant, and SVM operations) in assessing new samples. Without categorizing channels according to spatial-related groups, classification performance improved (Table 4); yet, the feature indices lacked robustness among optimization operations. This may suggest the spurious optimization we were concerned about.

**Table 4:** Summary of optimum features without spatially categorizing channel groups.

Characteristic	Condition	Optimization operation	Feature(s)	Specificity	Sensitivity	Accuracy
ΔC_O2Hb_ activation	Post-MPH	Simple	CH 37, 43, 44	86 ± 1.4%	91 ± 1.7%	84%
	medication	OR operation	CH 32, 37, 39, 41, 44 (Axis 1) CH 42 (Axis 2)	86 ± 1.4%	100%	84%
		AND operation	CH 37, 41, 42, 44 (Axis 1) CH 32, 43 (Axis 2)	86 ± 1.4%	100%	84%
		Linear discriminant	CH 32, 37, 42, 44 (Axis 1) CH 43 (Axis 2)	86 ± 1.7%	84 ± 7.2%	72%
		Quadratic discriminant	CH 32, 41, 44 (Axis 1) CH 39, 42 (Axis 2)	89 ± 2.1%	100%	84%
		Support vector machine	CH 37, 39, 41, 44 (Axis 1) CH 43 (Axis 2)	99 ± 2.5%	87 ± 6.3%	69%

**Figure 7:**
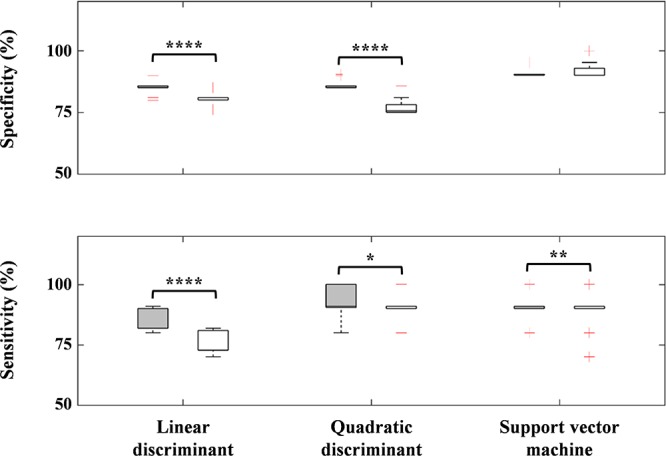
Performance differences between the two- (gray-filled boxplots) and three-axis (void-filled boxplots) feature thresholds using linear discriminant, quadratic discriminant, and SVM operations for specificity and sensitivity parameters. ^∗^*p* < 0.05; ^∗∗^*p* < 0.01; ^∗∗∗∗^*p* < 0.0001 for paired-sample *t*-test.

We found three robust regions (i.e., right MFG, ANG, PrCG) to distinguish between the ADHD and ASD-comorbid ADHD groups. For further confirmation, we evaluated classification performance using the three-axis feature thresholds (each region as an axis) for linear discriminant, quadratic discriminant, and SVM operations (Figure 7). However, we observed either insignificant improvement or significantly decreased performance compared to the two-axis feature thresholds. This might be an important clue in determining the strong relationship between right MFG and right ANG. An interpretation of the MFG-ANG relation is the attentive frontal-parietal network (Chochon et al., 1999; Peers et al., 2005; Rivera et al., 2005). Even though the GNG task is an inhibition-control task, attentive components are also involved in this task such as recognizing go cues and frequently corresponding to the rate of omission-commission errors (attentional impulsivity) and response time (Murphy et al., 1999; Keilp et al., 2005; Reynolds et al., 2006). PrCG, i.e., primary motor area activation, likely explains the ability of motor movement in the arms and hands. Suskauer et al. (2008) reported the differences between TD and ADHD children in motor activation (i.e., pre-supplementary motor area) during the GNG task. Abnormality in PrCG connectivity was also observed in ASD children (Nebel et al., 2014). However, as we discussed above, comorbidity may complicate the interpretation of non-modulated activation in right MFG-ANG and right PrCG regions after MPH administration for ASD-comorbid ADHD children. Nevertheless, we could still confirm the feasibility of MPH-evoked ΔC_O2Hb_ activation as an advanced feature.

### Limitations

We encountered three limitations in this study. First, the sample number was limited and imbalanced. The obtained feature should be validated in new datasets with a larger sample number to anticipate the inflated group variances as the sample number increases. However, given the absence of neuroimaging-based classification involving ASD-comorbid ADHD children, this study should provide an initial step in encouraging further studies. Second, as we mentioned above, the relationships among behavioral performance, personal traits, symptomal-disorder severity level, and brain activation are still unknown. However, it is necessary to interpret individual neuropathophysiology in ADHD and ASD-comorbid ADHD children. Future research may address this issue by using multiple factor analysis. Third, the detailed mechanism and pharmacological effect on brain activation in ASD-comorbid ADHD children are still unknown. Further investigation is required to interpret the divergence of MPH-evoked response in both groups. The evaluation of medication efficacy over time should be addressed to assess neuromodulation and neuroadaptability to medication.

## Conclusion

We investigated the effective biomarkers as features to differentially distinguish between ADHD and ASD-comorbid ADHD children. The characteristics of features were optimized and cross-validated. The most optimum feature was selected on the basis of distinct MPH-evoked response on ΔC_O2Hb_ activation which ADHD children presenting increased activation; yet, ASD-comorbid children presented hypoactivation in the right hemisphere. This suggests the feasibility of implementing fNIRS measurement, the GNG task, and the current features as clinically differential diagnostic biomarkers.

## Author Contributions

YM, MN, TI, and TT designed and performed the experiments. SS conceived the presented idea and performed the computation analysis. MK, TT, and ID verified the analysis. YM, AM, TY, and ID supervised the finding of this work. All authors discussed the results and contributed to the final manuscript.

## Conflict of Interest Statement

The authors declare that the research was conducted in the absence of any commercial or financial relationships that could be construed as a potential conflict of interest.
